# Spätzle processing enzyme is required to activate dorsal switch protein 1 induced Toll immune signalling pathway in *Tenebrio molitor*

**DOI:** 10.1371/journal.pone.0291976

**Published:** 2023-09-21

**Authors:** Md. Mahi Imam Mollah

**Affiliations:** 1 Department of Plant Medicals, College of Life Sciences, Andong National University, Andong, Republic of Korea; 2 Department of Entomology, Faculty of Agriculture, Patuakhali Science and Technology University, Patuakhali, Bangladesh; Universidade Federal do Rio de Janeiro, BRAZIL

## Abstract

Dorsal switch protein 1 (DSP1) acts as a damage-associated molecular pattern (DAMP) molecule to activate immune responses in *Tenebrio molitor*. From a previous study in *Spodoptera exigua*, we found that DSP1 activates Toll immune signalling pathway to induce immune responses by melanisation, PLA_2_ activity and AMP synthesis. However, the target site of DSP1 in this pathway remains unknown. The objective of this study was to determine the role of spätzle processing enzyme in the DSP1 induced toll immune signalling pathway. To address this, we analyzed spätzle processing enzyme (*Tm-SPE*) of the three-step serine protease cascade of *T*. *molitor* Toll pathway. *Tm-SPE* expressed in all developmental stages and larval tissues. Upon immune challenge, its expression levels were upregulated but significantly reduced after RNA interference (RNAi). In addition, the induction of immune responses upon immune challenge or recombinant DSP1 injection was significantly increased. Loss of function using RNA interference revealed that the *Tm-SPE* is involved in connecting DSP1 induced immune responses like hemocyte nodule formation, phenoloxidase (PO) activity, phospholipase A_2_ (PLA_2_) activity and antimicrobial peptide (AMP) synthesis. These suggest that *Tm-SPE* controls the DSP1 induced activation of Toll immune signalling pathway required for both cellular and humoral immune responses. However, to confirm the target molecule of DSP1 in three-step proteolytic cascade, we have to check other upstream serine proteases like Spatzle activating enzyme (SAE) or modular serine protease (MSP).

## Introduction

High mobility group box 1 (HMGB1) is a ubiquitously expressed, highly conserved ‘chromatin-associated protein’ in vertebrates [1]. At normal condition, it remains in nucleus to contribute gene expression, DNA repair, chromatin rearrangement etc. [2–4] while released from nucleus under stress to acts as a damage-associated molecular pattern (DAMP) that activate innate immune responses by interacting with pattern recognition receptors like Toll-like receptors (TLRs), receptor for advanced glycation end products (RAGE) etc. [5]. Dorsal Switch Protein 1 (DSP1) is the insect homolog of vertebrate HMGB1 first introduced in *Drosophila melanogaster* [6] as a transcriptional co-repressor and chromatin remodelling factor [7, 8]. DSP1 exhibits nuclear function like DNA binding [9], binding with *zen* and Rel to activate transcription in *Anophiles gambie* [10], regulation of *Sex combs reduced* (*Scr*), *Ultrabithorax (Ubx)* and *Abdominal-B* gene expression [8]. DSP1 also mediate immune responses upon pathogen infection in insects like *Spodoptera exigua* [11] and *Tenebrio molitor* [12] by activating eicosanoid that mediate cellular and humoral immune responses in insects [13]. Following Toll signalling pathway, DSP1 activate phospholipase A_2_ (PLA_2_), an enzyme that catalyse phospholipids to biosynthetic precursor(s) eicosanoids [14].

Toll immune signalling pathway in *Drosophila* is induced by Gram-positive bacteria or fungi and begins with the recognition of lysine-type peptidoglycan (PG) that triggers activation of the serine protease (SP) cascade leading to form active Spätzle [15, 16]. In *Tenebrio molitor*, SP cascade consists of three SPs [17, 18]. The initiating SP is a modular serine protease (modSP) which binds to the lysine-type PG recognition complex and activates the second SP, Spätzle activating enzyme (SAE), which in turn activates the last SP, Spätzle processing enzyme (SPE) [17, 19]. Inactive pro-Spätzle is cleaved by SPE to form active Spätzle, which binds to Toll receptor and triggers its specific immune signalling pathway [20, 21]. SPE also cleaves inactive prophenoloxidase (PPO) to active phenoloxidase (PO) [22] that leads to melanisation on the surface of bacteria to kill them. Thus, SPE mediates Toll immune signalling pathway to produce antimicrobial peptides (AMPs) and the melanisation immune responses [19].

DSP1 was predicted and known to mediate immune responses in *T*. *molitor* [12] and showed that DSP1 up-regulated gene expression of some AMPs and activated PLA_2_, PO and nodulation in response to Gram-positive bacterium containing Lys-type PG. A previous study in *S*. *exigua* reported that DSP1 activate Toll immune signaling pathways for immune mediation [14]. Another study in *T*. *molitor* reported that the Toll pathway includes a three-step proteolytic cascade such as modSP, SAE and SPE for mediating immune responses induced by peptidoglycans [17]. But we do not know the role of SPE or other upstream serine protease in DSP1 induced Toll pathway. Thus, present study aimed to know the involvement of SPE among the three-serine protease of *T*. *molitor* Toll pathway in mediating DSP1 induced immune responses. Present study showed that SPE control the immune responses induced by DSP1. This suggests that DSP1 depends on the SPE to activate Toll immune signalling pathway in *T*. *molitor*.

## Materials and methods

### Insect rearing and bacteria culture

Larvae of *Tenebrio molitor* (*T*. *molitor*) were collected from Bio Utility, Inc. (Andong, Korea) and grown on a diet of wheat bran [23] at 25 ± 2°C temperature; 60 ± 5% relative humidity and 16:8 h (L:D) photoperiod and underwent 12 larval instars (L1-L12) to become adult. Adults were provided wheat bran supplemented with cabbage [24]. Gram-positive bacterium, *Enterococcus mundtii* (*E*. *mundtii*) and Gram-negative *Xenorhabdus hominickii* (*X*. *hominickii*) were cultured overnight in tryptic soy broth (TSB) (Difco, Sparks, MD, USA) medium at 28°C with shaking at 180 rpm. For immune challenge, bacteria culture was centrifuged at 10,000 × *g* for 5 min, then the pellet was resuspended in sterile distilled water or PBS as needed. Bacterial cells were counted under a phase contrast microscope (BX41, Olympus, Tokyo, Japan) using a hemocytometer (Neubauer improved bright line, Superior Marienfeld, Lauda-Konigshofen, Germany). Bacteria was injected into L6 larvae (1.8 × 10^5^ cells/larva) with a microsyringe (Hamilton, Reno, NV, USA).

### Chemicals

L-3,4-dihydroxyphenylalanine (DOPA) was purchased from Sigma-Aldrich Korea (Seoul, Korea) and dissolved in 100 mM PBS before assay. Anticoagulant buffer (ACB) needed to prevent hemocyte coagulation was prepared with 186 mM NaCl, 17 mM Na_2_EDTA and 41 mM citric acid adjusting the pH at 4.5. Metafectene Pro, a transfection reagent, needed to mix with dsRNA was purchased from Biontex (Plannegg, Germany). Phosphoric acid (100 mM) was used to prepare phosphate-buffered saline (PBS, pH 7.4). Purified recombinant DSP1 (rDSP1) of *Spodoptera exigua* was prepared in a previous study [11] and used in this study.

### Bioinformatics and sequence analysis

*Tm-SPE* was predicted from the whole genome shotgun contig (GenBank accession number: JABDTM010027791.1) using the *Drosophila* SPE as bait. The resulting sequence was subjected to further analysis with FGENESH Soft berry program (http://www.softberry.com/berry.phtml) to obtain an open read frame (ORF). ORF sequences were deposited to GenBank (accession number: MZ 190162.1). Phylogenetic analyses were performed using MEGA6 and Clustal W programs from EMBL-EBI (www.ebi.ac.uk) using Neighbor-joining method and the used genes accession numbers are presented in S2 Table. Bootstrapping values were obtained with 1,000 repetitions to support branching and clustering. Protein domains were predicted using SMART search program (http://smart.embl-heidelberg.de/).

### RNA extraction and RT-qPCR

Total RNAs from different developmental stages were extracted using 50 eggs, 20 L1 larvae, 10 L2 larvae, 2 L6 larvae, 1 L12 larva, one pupa, and one adult per replication. To extract total RNAs from different tissues, L6 larvae were used. Hemolymph from larvae was collected in ACB by cutting the abdominal tip. The collected hemolymph was centrifuged at 500 × *g* for 5 min. The resulting hemocyte pellet was used to extract total RNA. The remaining body after hemolymph collection was used to isolate fat body, midgut, and epidermis. Trizol reagent (Invitrogen, Carlsbad, CA, USA) was used for total RNAs extraction. After DNase treatment, 1 μg of total RNA was used to synthesize first-strand cDNAs using RT-Premix oligo-dT following [12]. Resulting cDNAs were used for PCR amplification with gene-specific primers (S1 Table). RT-qPCR was conducted using a Step One Real-Time PCR System (Applied Biosystem, Marsiling, Singapore) following the conditions: 95°C for 10 min for initial heat followed by 40 cycles of 95°C for 30 sec, different annealing temperature for 30 sec and 72°C for 30 sec. *T*. *molitor* specific gene, *L27a*, was used as reference gene [23]. Quantitative analysis was done with a comparative CT method [25] using the reference gene for normalization to estimate mRNA expression levels. Each treatment was replicated three times. Each time with three independent samples.

### RNA interference (RNAi) of *Tm-SPE* expression

Gene-specific primers (S1 Table) containing T7 promoter sequence (5´-TAATACGACTCACTATAGGGAGA-3´) at the 5´ ends were used to amplify template DNA. The resulting PCR product was used to synthesize double-stranded RNA (dsRNA) encoding *Tm-SPE* (dsSPE) using a MEGAscript RNAi kit (Ambion, Austin, TX, USA) at 37°C for 4 h. Synthesized single-stranded RNAs in both directions were allowed to anneal at 25°C after heat treatment at 75°C for 5 min. Control dsRNA (dsCON) was synthesized from a viral gene, *CpBV302* [26]. dsRNAs were mixed with a transfection reagent Metafectene PRO at a 1:1 (v/v) ratio and then incubated at 25°C for 30 minutes to allow liposome formation that increases RNAi efficiency [27]. One μg of dsRNA for each was injected into L6 using 10 μL Hamilton microsyringe. The RNAi efficiency was determined by RT-qPCR against *Tm-SPE* expression at 24, 48, 72 and 96 h post-injection (PI).

### Nodulation assay

Nodule formation assay was analysed to investigate the cellular immune response against *E*. *mundtii* or rDSP1 following [28]. Heat-killed *E*. *mundtii* (1.8 × 10^5^ cells/larva) was injected to L6 larvae through the pleuron using 10 μL Hamilton microsyringe at 24 h of dsRNA injection to induce hemocyte nodules. Number of nodules formed by *T*. *molitor* in the hemocoel as cellular immune response against bacterial challenge were counted. To rescue RNAi effect, recombinant DSP1 (rDSP1) was injected (600 ng/larva) along with bacterial challenge. After 8h incubation at 25°C, treated larvae were dissected under a stereomicroscope (Stemi SV 11, ZEISS, Jena, Germany). Nodules on the fat body were counted after removing the gut. Each treatment was replicated three times. Each time with five larvae.

### Phospholipase A_2_ (PLA_2_) assay

Both sPLA_2_ and cPLA_2_ enzyme activities in treated larvae samples were analyzed as described previously [12] using PLA_2_ Assay Kit (Cayman Chemical, Ann Arbor, MI, USA). For induction of PLA_2_ enzyme activity, L6 larvae were injected with 2.2 × 10^5^ cells of heat-killed *E*. *mundtii*. *Tm-SPE* was silenced using dsRNA specific to Tm-SPE by injecting 24 h before bacteria and rDSP1 injection. Hemolymph and fat body samples from treated larvae were collected at 8 h after the bacteria and rDSP1 injection. Plasma was separated from hemocyte by spinning at 500 × g for 5 min. Plasma and fat body samples were used for measuring sPLA_2_ and cPLA_2_ activity, respectively. Each treatment was replicated three times. Each time with three independent samples.

### Phenoloxidase (PO) enzyme assays

PO activity in plasma of treated larvae was determined using DOPA substrate as described by [12]. To induce PO activity, L6 larvae were challenged with *E*. *mundtii* dissolved in PBS before 8h of sample (plasma) collection. Naïve larvae were injected with sterile PBS. The total reaction volume (200 μL) consisted of 180 μL of 10 mM DOPA in PBS and 20 μL of plasma collected from treated larvae. Absorbance was measured at 490 nm using a VICTOR multi label Plate reader (PerkinElmer, Waltham, MA, USA). PO activity was expressed as ΔABS/min/μL of plasma. Each treatment was replicated three times. Each time with three independent samples.

### AMP genes expression pattern

Whole body tissues were evaluated for AMP genes expression that represent humoral immune responses. AMP genes expression in L6 larvae were determined after injecting 2.2 × 10^5^ cells of heat killed *E*. *mundtii* per larvae at 24 h post injection of dsRNAs. To rescue the RNAi effect, rDSP1 (600 ng/larva) was injected to the larvae along with the bacterial cells. At 8 h of bacteria injection, samples were collected to be used for RNA extraction. Three larvae were used for sample collection and subsequent RNA extraction, for each replication. AMP genes expression levels were assessed by RT-qPCR using gene specific primers (S1 Table) using *L27a* as reference gene. Each treatment was replicated three times. Each time with three independent samples.

### Bioassay of RNAi-treated *T*. *molitor* against *E*. *mundtii (Em)* and *X*. *hominickii (Xh)*

To assess the virulence of Gram-positive *E*. *mundtii* and Gram-negative *X*. *hominickii* on RNAi-treated *T*. *molitor*, L6 larvae were injected with 1 μg of dsRNA specific to *Tm-SPE* along with transfection agent, metafectene at 1:1 ratio. For control, larvae were injected with control dsRNA along with metafectene. After 24 h of dsRNA injection when the *Tm-SPE* is mostly silenced, same larvae were injected with either *E*. *mundtii* (2.2 x 10^5^ cells/larvae) or *X*. *hominickii* (1.8 x 10^5^ cells/larvae) to observe their virulence against *T*. *molitor* larvae. For injection, overnight grown bacteria were spined for 5 minute and the pallets were dissolved in PBS. The vial containing the bacterial suspension was vortexed very well to confirm almost equal number of bacterial insertion using Hamilton syringe. The treated larvae were incubated at room temperature (25 ± 2°C) providing enough diet for another 7 days under the rearing conditions and counted the mortality at each 24 h. To check the immune association of DSP1, rDSP1 was injected along with gram positive *E*. *mundtii*. Each treatment was replicated three times. Each replication consists of ten larvae.

### Statistical analysis

All data for continuous variables were subjected to one-way analysis of variance (ANOVA) using PROG GLM in SAS program [29]. Mortality data were subjected to arcsine transformation and used for ANOVA. Means were compared with the least significant difference (LSD) test at Type I error = 0.05. Median lethal dose (LD_50_) was obtained from Probit analysis using EPA Probit Analysis Program, ver. 1.5 (U.S. Environmental Protection Agency, USA). Each treatment was replicated three times.

## Results

### Prediction of spätzle processing enzyme (*Tm‐SPE*) from *T*. *molitor* transcriptome

**Fig 1:**
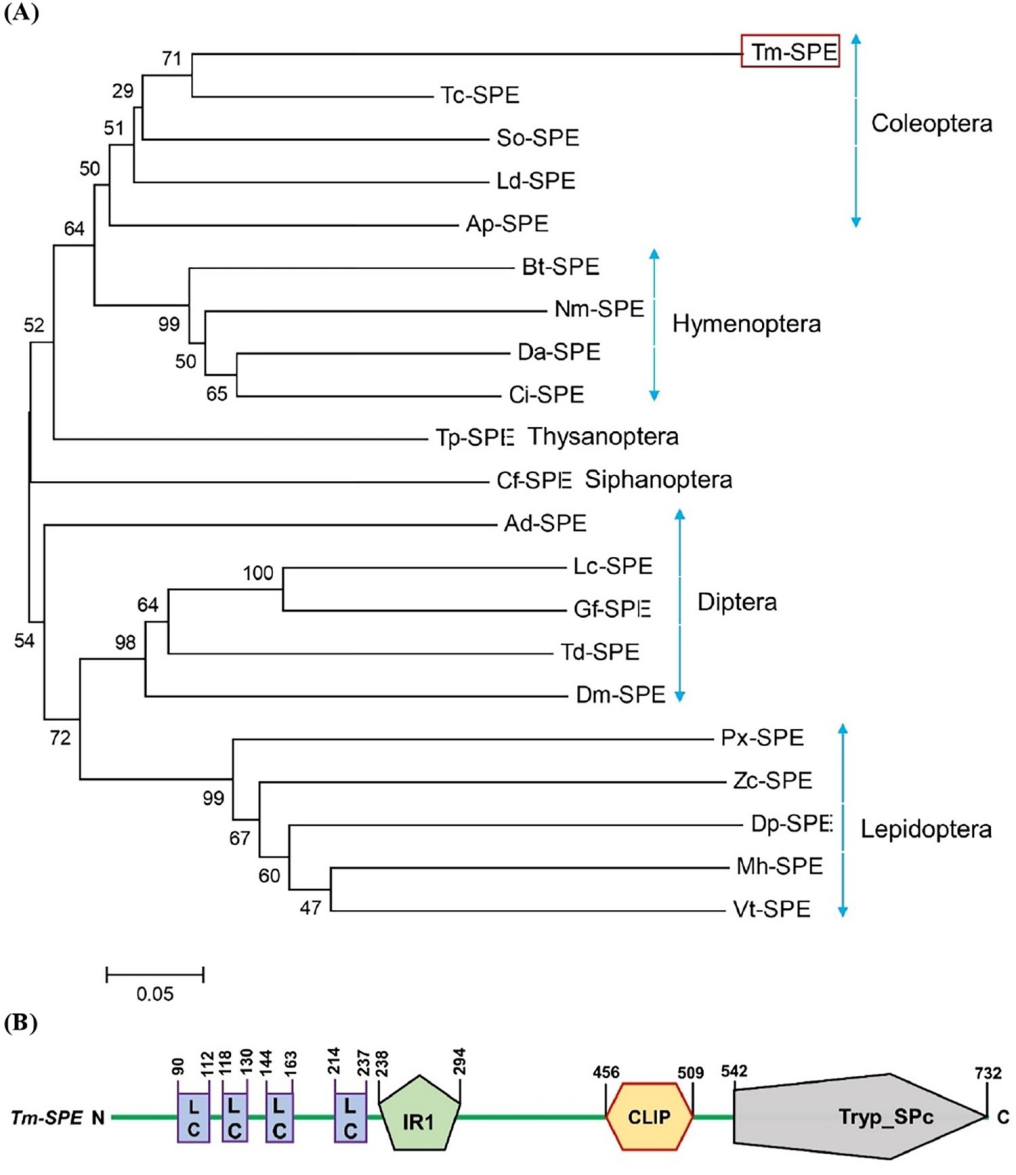
Molecular characterization of *T*. *molitor* Spätzle-Processing Enzyme (*Tm-SPE*). (**A**) Phylogenetic analysis of *Tm-SPE* with other insect SPE genes using MEGA6 program with a Neighbor-joining method. Bootstrapping values were obtained with 1,000 repetitions to support branching and clustering. Amino acid sequences of SPE were retrieved from GenBank with accession numbers shown in S2 Table. (**B**) Domain analysis of *Tm-SPE*. Domains were predicted using Prosite (https://prosite.expasy.org/) and SMART protein (http://smart.embl-heidelberg.de/).

Tm‐SPE was predicted from a transcriptome (GenBank accession no. JABDTM010027791.1) by interrogation with *D*. *melanogaster* SPE sequence (GenBank accession no. NM_142911.4) as a query. It has 99.65 percent sequence similarity with previously predicted Tm-SPE [17] and was deposited to GenBank (Accession no. MZ 190162.1). Its ORF consists of 2217 bp encoding 738 amino acids. The predicted amino acid sequence of Tm‐SPE was compared to those of other insect SPE genes through a phylogenetic analysis where SPE genes of the insects of same order clustered together (Fig 1A). Tm‐SPE shared 52.77%, 49.40%, 32.68%, 36.91% and 36.68% amino acid sequence similarities with SPE genes of *Tribolium castaneum*, *Leptinotarsa decemlineata*, *Drosophila melanogaster*, *Plutella xylostella* and *Apis cerana cerana*, respectively. Phylogenetic analysis showed that *Tm‐SPE* was closely related to other coleopterans. *Tm‐SPE* comprised of Low Complexity (LC), Internal Repeat 1 (IR1), CLIP and Tryp-Spc domains (Fig 1B) whereas *Drosophila* SPE contains only Low Complexity (LC) and Tryp-Spc domains.

### Expression profile of *Tm-SPE* in *Tenebrio molitor*

**Fig 2:**
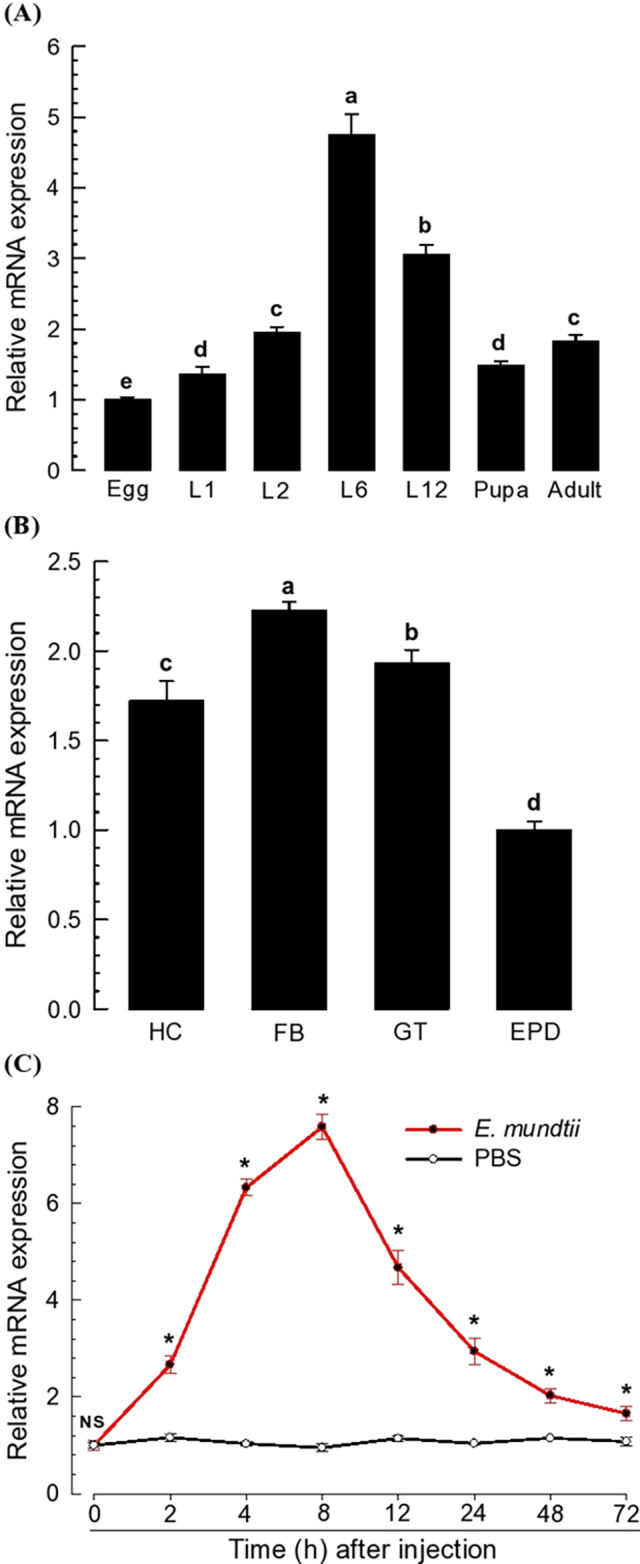
Expression profiles of *Tm-SPE*. (**A**) Expression patterns of *Tm-SPE* at different developmental stages: egg, some larval instar (‘L1, L2, L6, L12’), pupa and adult. (**B**) Expression patterns in different tissues of L6 larvae: hemocyte (‘HC’), fat body (‘FB’), midgut (‘GT’) and epidermis (‘EPD’). (**C**) Inducible expression of *Tm-SPE* in whole body sample of L6 larvae at different time intervals after bacterial infection. For bacterial challenge, heat-killed *E*. *mundtii* (‘Em’, 1.8 × 10^5^ cells/larva) were injected into each larva. For control, larvae were injected with PBS. Expression was analyzed with real-time qPCR and up-regulated expression levels were calculated as fold change of the lowest expressed. A ribosomal gene, *L27a*, was used as internal control [24]. Each treatment was replicated three times with independent samples preparation. Different letters above the standard deviation bar indicate significant differences among means at Type I error = 0.05 (LSD test).

*Tm-SPE* was expressed in all developmental stages ranging from egg to adult and showed high expression levels at larval stages, especially at L6. However, its expression levels were low at the egg and pupal stage than adult stage (Fig 2A). At L6, it was expressed in all tested tissues including hemocytes, fat body, gut, and epidermis. However, its expression levels were different among tissues, showing a high expression level in the fat body and low expression level in epidermis (Fig 2B). Basal expression levels were significantly upregulated following immune challenge with *E*. *mundtii* in whole body tissue where the maximum expression (more than 7-fold) was at 8 h of injection (Fig 2C). However, at very early stage (2 h of injection), expression level was around 3-fold. The upregulation of *Tm‐SPE* expression after bacterial challenge suggested its immunological function.

### RNAi of *Tm-SPE* and interruption of nodulation and PO activity

**Fig 3:**
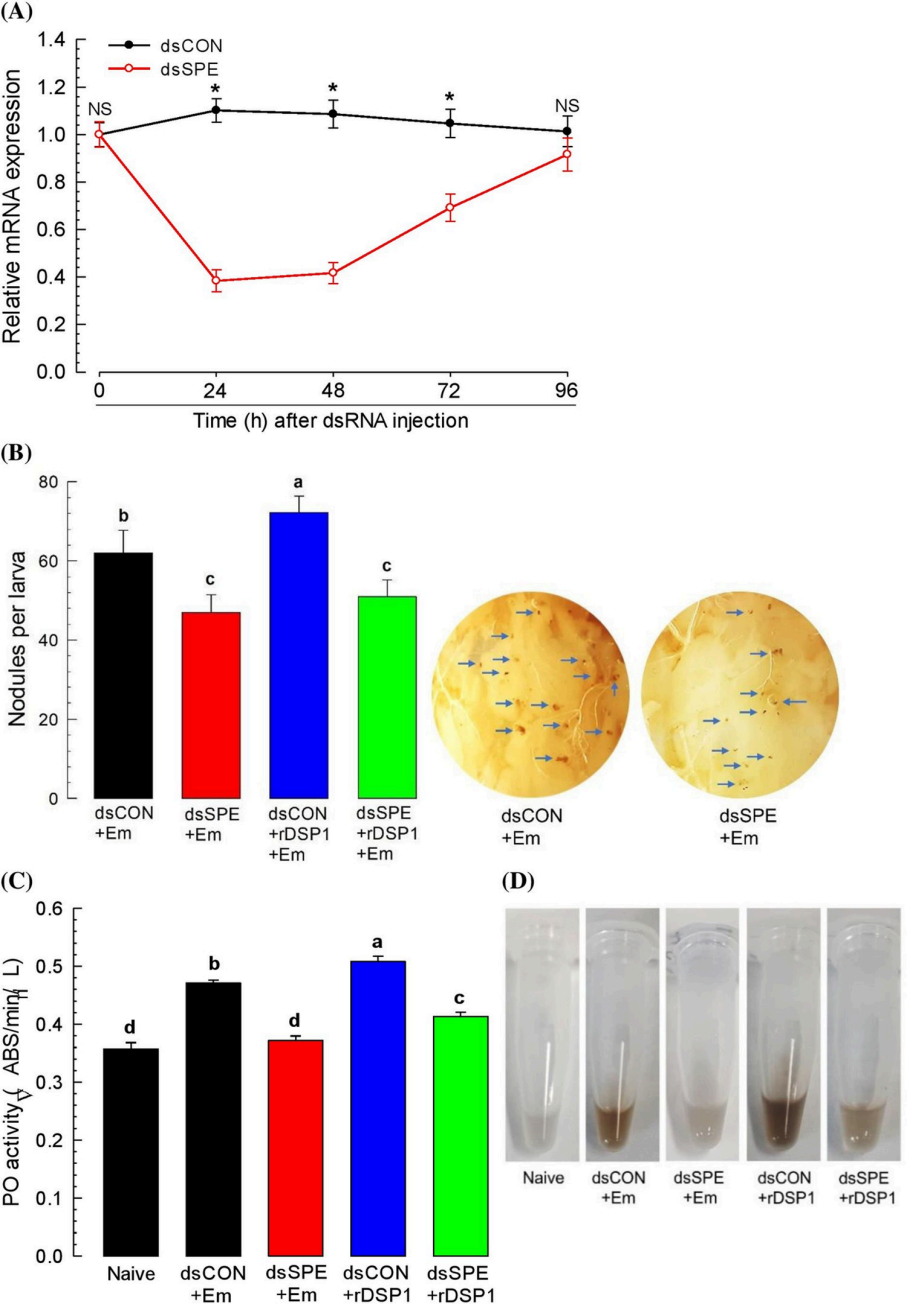
Role of SPE for the induction of immune responses by *E*. *mundtii* and rDSP1. (**A**) RNAi efficiencies of *Tm-SPE* gene by injecting gene-specific dsRNA (‘dsSPE’, 1 μg/larva) to L6 larvae. A viral gene, *CpBV302*, was used as a control dsRNA (‘dsCON’). (**B**) *E*. *mundtii* and rDSP1 induced mediation of hemocyte nodulation, (**C**) PO activity and (**D**) Pictorial representation of change in plasma due of PO activity. L6 larvae of *T*. *molitor* were injected with *E*. *mundtii* (2.2 x 10^5^ cells/larvae) dissolved in PBS or *E*. *mundtii* (2.2 x 10^5^ cells/larvae) along with recombinant DSP1 (‘rDSP1, 0.6 μg/larva). At 8 h of *E*. *mundtii* or rDSP1 injection, nodules were counted in each larva by dissecting and plasma was collected for PO activity analysis. Naïve was injected with PBS. Knock-down of *Tm-SPE* expression was done by injecting dsRNA specific to *Tm-SPE* (1 μg/larva) into L6 larvae before 24 h of *E*. *mundtii* or rDSP1 injection. A viral gene, *CpBV302*, was used as a control dsRNA (‘dsCON’). Each treatment was replicated three times. Asterisks indicate significant differences between dsSPE and dsCON treated group at Type I error = 0.05 (LSD test). Different letters above standard deviation bars denotes significant difference among means at Type I error = 0.05 (LSD test).

Expression of *Tm-SPE* was suppressed by RNAi via injection of gene‐specific dsRNA. Such RNAi treatment significantly (*P* < 0.05) reduced the expression levels around 60 percent at 24 h of dsRNA post injection which continued up to 48 h and then starts to increase. Such reduced levels were recovered at 96 h PI (Fig 3A). These down regulation of *Tm-SPE* expression denotes the efficiency of RNAi of *Tm-SPE* gene.

To know the immune association of *Tm-SPE*, nodulation and PO activity was analyzed at both non-silenced and silenced condition of *Tm-SPE*. At non-suppressed condition, increased number of nodules (62 ± 5.70) formed in the insect hemocoel upon *E*. *mundtii* challenge which decreased (47 ± 4.47) significantly (*P* < 0.05) at the *Tm-SPE* silenced condition obtained from RNAi of *Tm-SPE* (Fig 3B). PO activity in larval plasma was also induced by *E*. *mundtii* which significantly (*P* < 0.05) reduced after RNAi of *Tm-SPE* (Fig 3C). To know the role of Tm-SPE in DSP1 induced immune mediation, nodulation and PO activity was assessed in recombinant DSP1 challenged larvae at both *Tm-SPE* silenced and non-silenced condition. For both the cases, induction was very high and significantly (*P* < 0.05) more compared to naïve and even *E*. *mundtii* which decreased significantly (*P* < 0.05) at silenced condition of *Tm-SPE* gene (Fig 3B and 3C). This result thus denotes that both *E*. *mundtii* and rDSP1 depends on SPE for the induction of hemocyte nodulation and PO activity.

### Induction of PLA_2_ activity in *T*. *molitor* by *E*. *mundtii* or rDSP1

**Fig 4:**
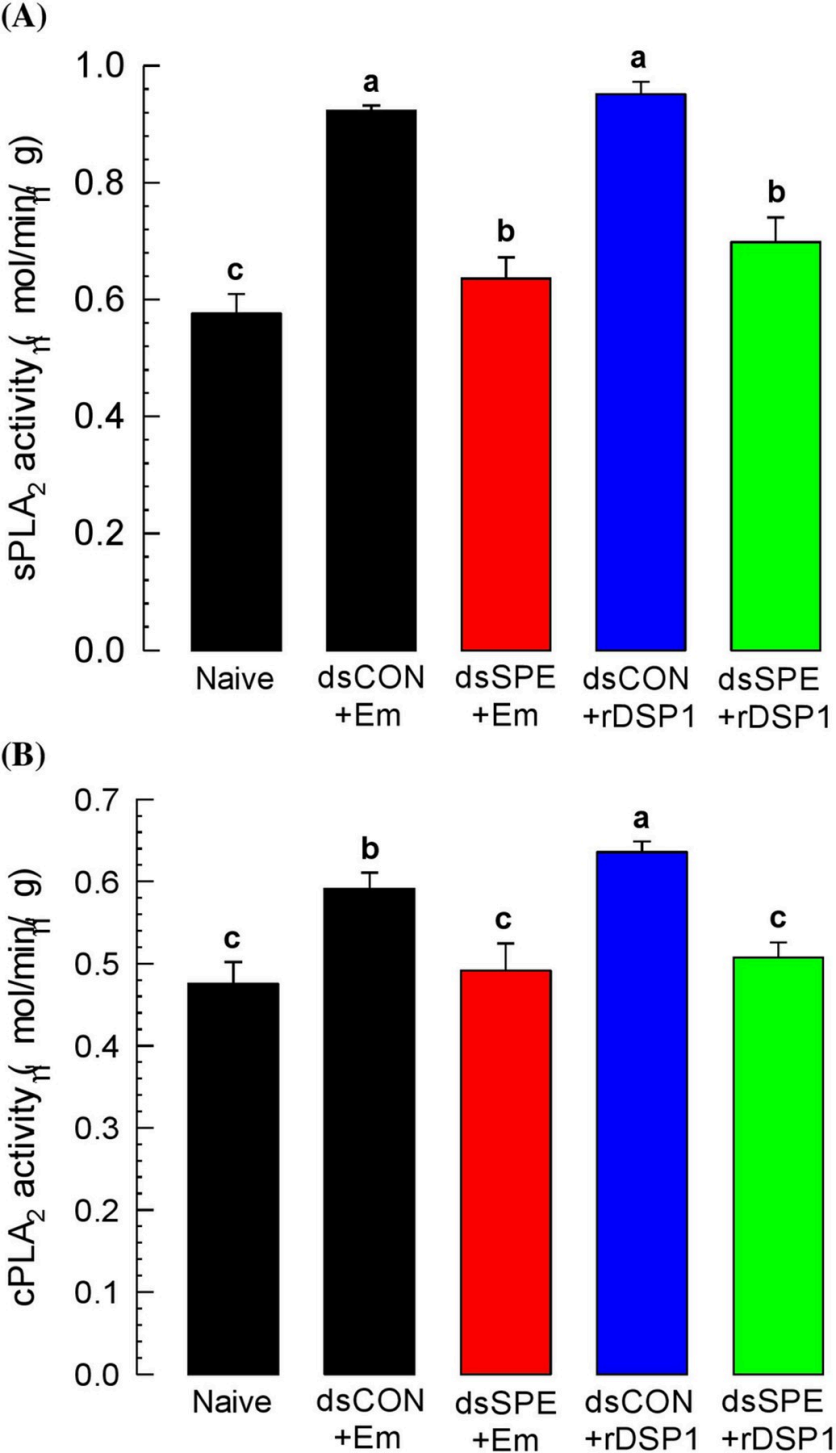
Functional assay of spätzle processing enzyme (*Tm-SPE*) for PLA_2_ activity mediation. (**A**) Mediation of sPLA_2_ activity induced by bacteria and rDSP1 via SPE. (**B**) Mediation of cPLA_2_ activity by bacteria and rDSP1 via SPE. L6 larvae were injected with *E*. *mundtii* (2.2 x 10^5^ cells/larvae) or recombinant DSP1 (‘rDSP1’, 0.6 μg/larva). Naïve were injected with PBS. dsRNA specific to SPE or dsCON was injected 24 h before bacteria or rDSP1 injection. At 8 h after *E*. *mundtii* or rDSP1 injection, Fatbody and plasma was collected to determine cPLA_2_ and sPLA_2_ activity, respectively. dsRNA was made from a viral gene (*CpBV302*). Each treatment was replicated three times. Different letters above standard deviation bars denotes significant difference among means at Type I error = 0.05 (LSD test).

Upregulation of *Tm-SPE* after immune change indicate its immune association. To validate this, both sPLA_2_ and cPLA_2_ activity was analysed upon immune challenge. Induction of sPLA_2_ activity was significant (*P* < 0.05) upon *E*. *mundtii* challenge which was reduced after RNAi of *Tm-SPE* (Fig 4A). Similarly, cPLA_2_ is also induced significantly by *E*. *mundtii* but RNAi of *Tm-SPE* suppressed it (Fig 4B). To see the immune mediation by DSP1, recombinant DSP1 was injected into the larvae of *T*. *molitor*. rDSP1 injected larval plasma showed significantly higher sPLA_2_ (Fig 4A) and cPLA_2_ activity (Fig 4B) which was reduced significantly after RNAi of *Tm-SPE* gene. This result suggests that rDSP1 can activate both the sPLA_2_ and cPLA_2_ activity similar to *E*. *mundtii* depending on SPE for mediation of PLA_2_ activity.

### Induction of AMP genes expression in *T*. *molitor* by bacteria or rDSP1

**Fig 5:**
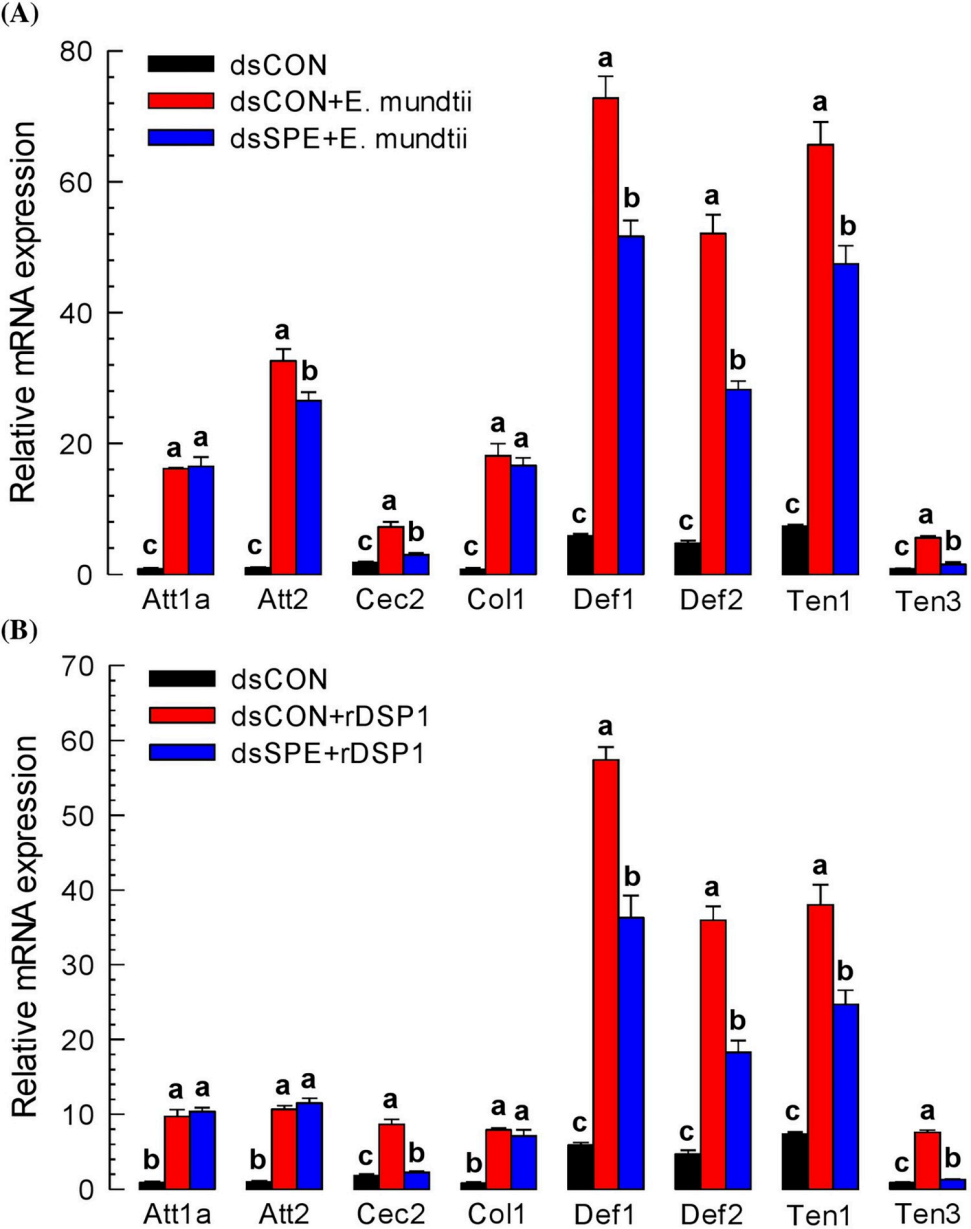
Expression pattern of AMP genes in *T*. *molitor*. (**A**) Mediation of AMP genes expression via SPE upon *E*. *mundtii* challenge (**B**) Mediation of AMP genes expression via SPE upon rDSP1 challenge. For AMP genes expression upon bacterial challenge, *E*. *mundtii* were dissolved in PBS (‘Em’, 1.8 × 10^5^ cells/larva) and for rDSP1 challenge, purified rDSP1 (0.6 μg/larva) was injected into L6 larva. For analysing expression of Attachin1a (‘*Att1a*’), Attachin2 (‘*Att2*’), Cecropin2 (‘*Cec2*’), Coleoptericin1 (‘*Col1*’), Defensin1 (‘*Def1*’), Defensin2 (‘*Def2*’), Tenesin1 (‘*Ten1*’) and Tenesin3 (‘*Ten3*’) AMP genes, whole body samples were collected after 8 h of bacterial or rDSP1 challenge for RNA extraction. For knockdown of Tm-SPE gene, dsRNA specific to Tm-SPE was injected 24 h before *E*. *mundtii* or rDSP1 injection. For control, dsRNA made from a viral gene (*CpBV302*) was injected. Each treatment was replicated three times with independent tissue preparations. Different letters above the bar indicate significant differences among means at Type I error = 0.05 (LSD test).

Under induced *Tm-SPE* expression, immunity‐associated AMP gene’s synthesis was assessed. AMP synthesis in the larval tissues was significantly (*P* < 0.05) increased after bacterial challenge. Eight AMP genes specific to *T*. *molitor*, such as Attachin1a (*Att1a*), Attachin2 (*Att2*), Cecropin2 (*Cec2*), Coleoptericin1 (*Col1*), Defensin1(*Def1*), Defensin2(*Def2*), Tenesin1 (*Ten1*) and Tenesin3 (*Ten3*) were analysed for their expression up on bacterial and rDSP1 challenge. All the AMP genes expressed in naïve or control tissue which strongly upregulated up on *E*. *mundtii* injection and the numerical value of AMP expression especially for *Atta1a*, *Att2*, *Col1*, *Def1*, *Def2* and *Ten1* convey the message (Fig 5A). After RNAi of *Tm-SPE*, expression of AMP genes decreased for *Att2*, *Cec2*, *Def1*, *Def2*, *Ten1* and *Ten3* which indicate that *E*. *mundtii* induces the expression of these AMP genes. For rDSP1 induced AMP genes expression, similar trend was observed. After RNAi of *Tm-SPE*, expression of *Cec2*, *Def1*, *Def2*, *Ten1* and *Ten3* reduced significantly (*P* < 0.05) (Fig 5B). This result concludes that *Cec2*, *Def1*, *Def2*, *Ten1* and *Ten3* AMP genes are activated or controlled by rDSP1.

### Effect of spätzle processing enzyme (SPE) on susceptibility of *T*. *molitor*

**Fig 6:**
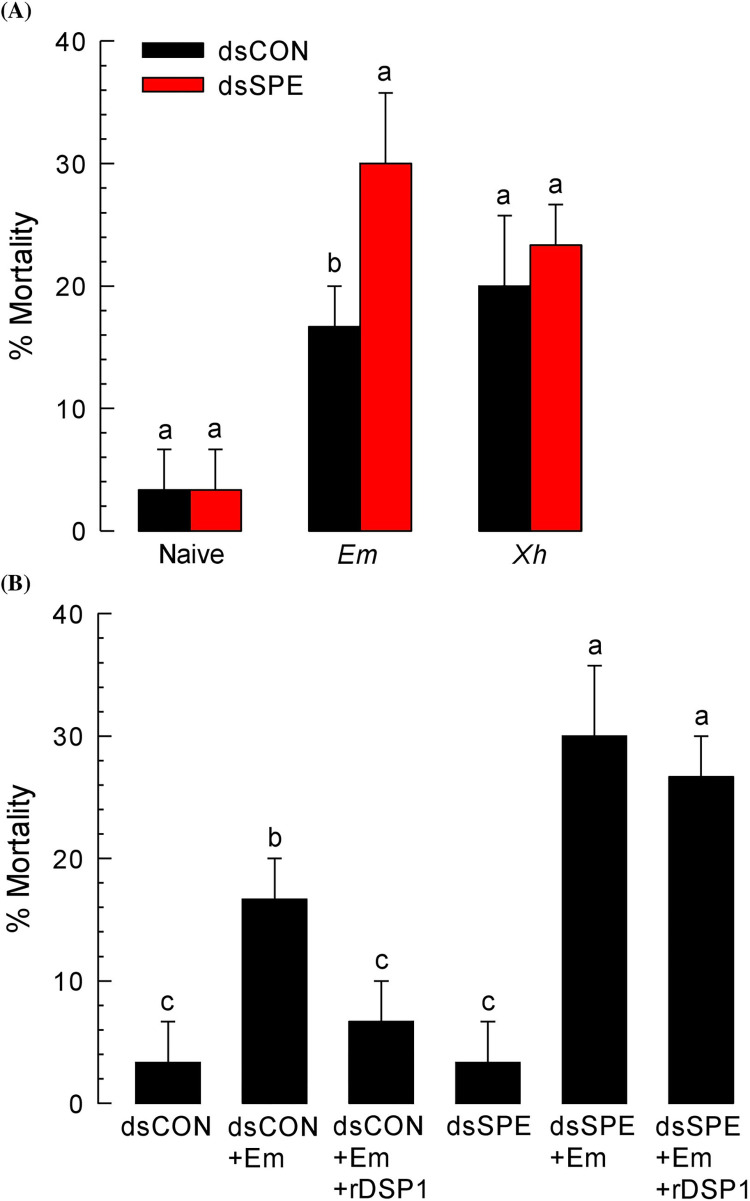
Comparative virulence analysis of *E*. *mundtii* (‘*Em*’) and *X*. *hominickii* (‘*Xh*’) bacteria against *T*. *molitor* larvae. **(A)** Individual RNAi treatment targeting SPE gene and (**B**) Immune induction by rDSP1 to increase survivability. For survivability test, Gram positive *E*. *mundtii* (3 x 10^4^ cells/larva) and Gram-negative *X*. *hominickii* (2.5 x 10^4^ cells/larva) were injected to L6 larvae of *T*. *molitor* already (24 h before) injected with dsSPE and dsCON (1 μg/larva). dsCON was made from a viral gene *CpBV302*. For immune mediation test of rDSP1, at 24 h post injection of dsRNA, *E*. *mundtii* (3 x10^5^ cells/larva) or *E*. *mundtii* along with rDSP1 (0.6 μg/larva) was injected to larvae with a Hamilton micro syringe. Mortality was recorded up to 7 days after treatment (‘DAT’). Each treatment was replicated three times and each replication used 10 larvae. Different letters above standard deviation bars denote significant difference among means at Type I error = 0.05 (LSD test).

Toll-spätzle immune signaling pathway is activated by Gram-positive bacteria. To confirm the involvement of *Tm-SPE* in Toll pathway activation, we checked virulence of both Gram-positive (*E*. *mundtii*) and Gram-negative (*X*. *hominickii*) bacteria against *T*. *molitor* larvae after individual RNAi treatment with dsSPE. Control larvae were treated with dsCON. For control larvae, *E*. *mundtii* found less virulent compared to *X*. *hominickii* whereas *E*. *mundtii* showed more virulence than *X*. *hominickii* against dsSPE treated larvae (Fig 6A). This indicate that SPE of *T*. *molitor* Toll pathway is essential for immune activation in Gram positive *E*. *mundtii* bacterium. To confirm the immune role of DSP1, recombinant DSP1 was injected into *T*. *molitor* larvae which significantly (*P* < 0.05) decreased the mortality in control larvae but no significant change in SPE silenced larvae (Fig 6B). This suggested that DSP1 is dependent on SPE for immune mediation. This finding concludes that DSP1 depend on SPE to follow the Toll-spätzle pathway to induce the immune activation.

## Discussion

Dorsal switch protein 1 (DSP1), an insect homolog of vertebrate HMGB1 protein was characterized as a co-repressor of Dorsal protein in *Drosophila* [7]. Dorsal is one of the transcriptional activators from the Rel/NF-kB family [7]. In *Drosophila*, DSP1 also play role in embryo development, differentiation, and segmentation [6, 30, 31]. In mosquito, *Aedes aegypti*, another HMGB1 having high homologies with mammalian HMGB1 and *Drosophila* DSP1 [32] has been identified. This mosquito HMGB1 facilitate the binding of transcriptional factor ‘Rel’ to the nuclear factor kappa B (NF-kB) promoter for antiviral gene expression against a dengue viral infection [33]. A lepidopteran insect (*Plodia interpunctella*), apart from dipterans, also possesses HMGB1-like proteins [34]. Recent study revealed the DAMP role of DSP1 in lepidopteran *S*. *exigua* [11], coleopteran *T*. *molitor* [12] and dipteran *Aedes albopictus* [35] that mediate different immune responses like PLA_2_ activity, nodulation formation, AMP synthesis, PO activity etc. [11–13]. Subsequent study in *S*. *exigua* reports that DSP1 uses Toll-spätzle pathway for immune mediation [14]. But we do not know the role of SPE in this pathway. To address this, we coined this study in *T*. *molitor* to know the involvement of SPE in DSP1 induced immune mediation through Toll-spatzle pathway. For this, recombinant DSP1 was injected into the larvae to observe gain of function or additive immune responses and lose of function after RNAi of SPE.

Toll immune signalling pathway initiated with the recognition of lysine-type peptidoglycan (PG) triggering the activation of serine protease (SP) cascade needs to form active Spätzle [15]. This active Spatzle binds with Toll receptor and Toll signaling is activated. In *Tenebrio molitor*, SP cascade consists of three SPs [17], starting with modular serine protease (modSP) that binds to the lysine-type PG recognition complex in upstream and activate the second SP, Spätzle activating enzyme (SAE), which in turn activates Spätzle processing enzyme (SPE), the last SP [17, 19]. SPE cleave the inactive pro-Spätzle to form active Spätzle, which binds to Toll receptor and triggers its specific immune signalling pathway [21, 36]. In other direction, SPE cleaves inactive prophenoloxidase (PPO) to active phenoloxidase (PO) [22]. Thus, SPE mediates Toll immune signalling pathway to produce antimicrobial peptides (AMPs), PLA_2_ activity and the melanisation immune responses [19] including PO activity, nodulation etc. In this study, we found that Gram-positive *E*. *mundtii* and rDSP1 upregulated the expression of Attachin1a (‘*Att1a*’), Attachin2 (‘*Att2*’), Cecropin2 (‘*Cec2*’), Coleoptericin1 (‘*Col1*’), Defensin1 (‘*Def1*’), Defensin2 (‘*Def2*’), Tenesin1 (‘*Ten1*’) and Tenesin3 (‘*Ten3*’) AMP genes (Fig 5A). A previous study in *T*. *molitor* reported that *Att1*, *Att2*, *Cec* and *Def* was upregulated by both Gram-positive bacteria and rDSP1 [12]. Another study in *S*. *exigua* revealed that Apolipophorin (*Apol*), Attachin (*Att1*, *Att2*), Cecropin (*Cec*), Defensin (*Def*), Gallerimycin (*Gal*), Gloverin (*Glv*), Lysozyme (*Lyz*) etc. AMP genes were induced by *E*. *mundtii* [14]. Gram-positive *E*. *mundtii* and rDSP1 also increase the PLA_2_ activity (Fig 4A and 4C), PO activity (Fig 3C) and hemocyte nodule formation (Fig 3A). In contrast, RNAi of SPE gene interrupt the immune responses of AMP synthesis (Fig 5B), PLA_2_ activity (Fig 4B and 4D), PO activity (Fig 3D) and hemocyte nodulation (Fig 3B). This indicates that SPE control these immune responses. Interruption of these immune responses by RNAi of SPE lead to increased mortality of *T*. *molitor* larvae upon *E*. *mundtii* infection (Fig 6). However, in controlled condition, *X*. *hominickii* shows more virulence to *T*. *molitor* larvae because *X*. *hominickii* is an entomopathogenic bacterium that release different chemicals [28, 37] to host for immune suppression [38, 39], Toxemia or apoptosis [40] resulting host mortality. There was no significant difference in virulence of *X*. *hominickii* at *Tm-SPE* silenced condition indicating non-association of SPE to *X*. *hominickii* (Fig 6A). Thus, we can conclude that Spatzle processing enzyme (SPE) is essential for *E*. *mundtii* and DSP1 to activate Toll immune signaling pathway. To confirm the exact target of DSP1 protein in Toll pathway we need to check the upstream targets of SAE or modSP also.

**Fig 7:**
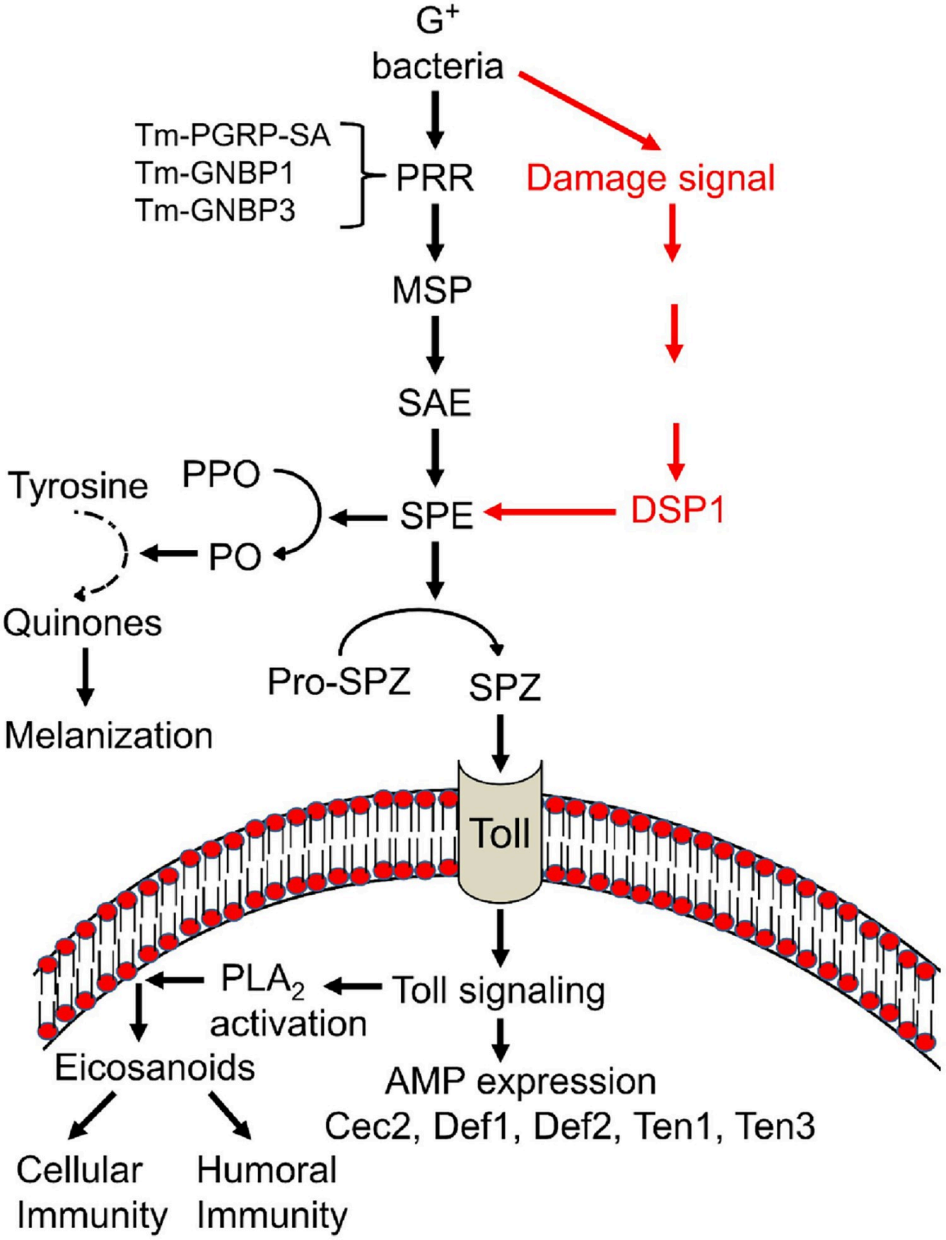
A working hypothesis of Tm-DSP1 for mediating immune responses via Toll-Spatzle (‘Spz’) signalling pathway. Upon challenge with gram positive bacteria including a non-entomopathogenic bacterium, *E*. *mundtii*, Tm-DSP1 is secreted to the plasma to activate serine protease (SP) cascade for activating phenoloxidase (‘PO’) and Spz. Activated PO can catalyze melanin formation to suppress the growth of pathogenic bacteria. Besides, activated Spz can bind to Tm-Toll receptor to activate PLA_2_ and the expression of antimicrobial peptide [Cecropine2 (‘Cec2’), Defencine1 (‘Def1’), Defencine2 (‘Def2’), Tenecine1 (‘Ten1’) and Tenecine3 (‘Ten3’)) genes. Activated PLA_2_ can catalyze eicosanoid biosynthesis to mediate cellular immune responses to defend bacterial infection along with AMPs.

In summary, DSP1 depends on SPE to activate the Toll signaling pathway required for innate immune response like nodulation, PLA_2_ and PO activity; AMP gene synthesis in *Tenebrio molitor* (Fig 7). However, all the AMP genes in *T*. *molitor* are not activated by this DSP1-Toll signaling pathway.

## Supporting information

S1 Table Primers used in this study with their information. (DOCX)

S2 Table GenBank accession number information of sequences used in Tm-SPE phylogeny analysis. (DOCX)

S1 Data Data of the Figures. (XLSX)
