# Supplementary material for: Spätzle processing enzyme is required to activate dorsal switch protein 1 induced Toll immune signalling pathway in Tenebrio molitor
Source: PLoS One. 2023 Sep 21;18(9):e0291976. doi: 10.1371/journal.pone.0291976 (PMC10513244; doi:10.1371/journal.pone.0291976)
Supplement: S1 Table — (DOCX) [file pone.0291976.s001.docx]

**Table S1.** Primers used in this study with their informations

| Genes | Primer sequences | Purpose |
| --- | --- | --- |
| *Tm-SPE* | 5´-GTAACAGTTGCGTTGAATCC-3´  5´-TGTCAACAAATATTGCACCG-3´ | RT-qPCR |
| *T7-Tm-SPE* | 5´-TAATACGACTCACTATAGGGAGA GTAACAGTTGCGTTGAATCC-3´  5´-TAATACGACTCACTATAGGGAGA TGTCAACAAATATTGCACCG-3´ | dsRNA preparation |
| Attacin1a | 5´-GAAACGAAATGGAAGGTGGA-3´  5´-TGCTTCGGCAGACAATACAG-3´ | AMP expression |
| Attacin2 | 5´-AACTGGGATATTCGCACGTC-3´  5´-CCCTCCGAAATGTCTGTTGT-3´ |  |
| Cecropin2 | 5´-TACTAGCAGCGCCAAAACCT-3´  5´-CTGGAACATTAGGCGGAGAA-3´ |  |
| Coleoptericin 1 | 5´-GGACAGAATGGTGGATGGTC-3´  5´-CTCCAACATTCCAGGTAGGC-3´ |  |
| Defensin1 | 5´-AAATCGAACAAGGCCAACAC-3´  5´-GCAAATGCAGACCCTCTTTC-3´ |  |
| Defensin2 | 5´-GGGATGCCTCATGAAGATGTAG-3´  5´-CCAATGCAAACACATTCGTC-3´ |  |
| Tenecin1 | 5´-CAGCTGAAGAAATCGAACAAGG-3´  5´-CAGACCCTCTTTCCGTTACAGT-3´ |  |
| Tenecin3 | 5´-GATTTGCTTGATTCTGGTGGTC-3´  5´-CTGATGGCCTCCTAAATGTCC-3´ |  |
| Tm-L27a | 5´-TCATCCTGAAGGCAAAGCTCCAGT-3´  5´-AGGTTGGTTAGGCAGGCACCTTTA-3´ | Endogenous control |
