# Supplementary material for: Spätzle processing enzyme is required to activate dorsal switch protein 1 induced Toll immune signalling pathway in Tenebrio molitor
Source: PLoS One. 2023 Sep 21;18(9):e0291976. doi: 10.1371/journal.pone.0291976 (PMC10513244; doi:10.1371/journal.pone.0291976)
Supplement: S2 Table — (DOCX) [file pone.0291976.s002.docx]

**S2 Table.** GenBank accession number information of sequences used in *Tm-SPE* phylogeny analysis.

| Gene name | Acronyms | GenBank Accession |
| --- | --- | --- |
| *Tenebrio molitor* SPE | *Tm-SPE* | MZ 190162.1 |
| *Tribolium castaneum* SPE | *Tc-SPE* | EFA07558.1 |
| *Sitophilus oryzae* SPE | *So-SPE* | XP_030764118.1 |
| *Leptinotarsa decemlineata* SPE | *Ld-SPE* | XP_023025567.1 |
| *Agrilus planipennis* SPE | *Ap-SPE* | XP_018332507.1 |
| *Belonocnema treatae* SPE | *Bt-SPE* | XP_033226882.1 |
| *Nomia melanderi* SPE | *Nm-SPE* | XP_031825659.1 |
| *Diachasma alloeum* SPE | *Da-SPE* | XP_015120009.1 |
| *Chelonus insularis* SPE | *Ci-SPE* | XP_034938681.1 |
| *Thrips palmi* SPE | *Tp-SPE* | XP_034237604.1 |
| *Ctenocephalides felis* SPE | *Cf-SPE* | XP_026480498.1 |
| *Anopheles darlingi* SPE | *Ad-SPE* | ETN59198.1 |
| *Lucilia cuprina* SPE | *Lc-SPE* | XP_023292431.1 |
| *Glossina fuscipes* SPE | *Gf-SPE* | XP_037884895.1 |
| *Teleopsis dalmanni* SPE | *Td-SPE* | XP_037953874.1 |
| *Drosophila melanogaster* SPE | *Dm-SPE* | NP_651168.1 |
| *Plutella xylostella* SPE | *Px-SPE* | XP_037963105.1 |
| *Zerene cesonia* SPE | *Zc-SPE* | XP_038206756.1 |
| *Danaus plexippus plexippus* SPE | *Dp-SPE* | XP_032521670.1 |
| *Maniola hyperantus* SPE | *Mh-SPE* | XP_034834258.1 |
| *Vanessa tameamea* SPE | *Vt-SPE* | XP_026494208.1 |
